# A study protocol for a preliminary randomised controlled trial assessing the acceptability and effectiveness of two eating disorders prevention interventions in Switzerland: The HEIDI BP-HW project

**DOI:** 10.1371/journal.pone.0259796

**Published:** 2021-11-15

**Authors:** Isabelle Carrard, Sophie Bucher Della Torre

**Affiliations:** Department of Nutrition and Dietetics, School of Health Sciences, University of Applied Sciences and Arts Western Switzerland (HES-SO), Geneva, Switzerland; Northumbria University Faculty of Health and Life Sciences, UNITED KINGDOM

## Abstract

Because of the serious consequences of eating disorders on young women’s lives and because of the lack of specialised care facilities, assessing and implementing evidence-based prevention interventions is necessary. Switzerland, like other Western countries, has high prevalence rates of eating disorders. However, no prevention interventions have been evaluated in this country so far. This paper presents the protocol of a preliminary study with the aim to evaluate the acceptability and effectiveness of two interventions, the Body Project (BP) and the Healthy Weight Program (HW), for female students from French-speaking Switzerland. These two interventions were chosen because they have been widely evaluated and they proved to be effective in various countries. They take place in groups and include four weekly sessions over one month. Because of the pandemic situation, the group sessions will take place online on an collaborative platform. The design is a three-arm randomised controlled study. Ninety female students aged 18–25 and presenting with at least moderate body dissatisfaction will be randomised into three groups: (1) one-month BP intervention, (2) one-month HW intervention, and (3) one-month waiting-list control group followed by the BP intervention. Assessments of body dissatisfaction, thin-ideal internalisation, dietary restraint, negative affect, and eating disorder psychopathology will be conducted before and after the interventions or waiting list and after a one-month follow-up. ANCOVA and ANOVA with repeated measures will be used to assess group differences and follow-up stability. Acceptability will be assessed with a questionnaire on participants’ satisfaction with the interventions, group discussion at the end of the intervention, and with participants’ rate of attendance to the group sessions. The study results will provide additional data on these two eating disorders prevention interventions and will suggest ways for their dissemination and further evaluation in Switzerland.

## Introduction

Eating disorders are serious mental illnesses with severe consequences on the somatic, psychological, and social levels [1,2]. The diagnoses described in the current version of the Diagnostic and Statistical Manual of Mental Disorders (DSM-5 [3]) include three main disorders: 1) anorexia nervosa, which is characterised by severe dietary restriction and a weight below a healthy body mass index (BMI); 2) bulimia nervosa, which is characterised by episodes of compulsive eating and compensatory behaviours for weight control; and 3) binge eating disorder, which also includes compulsive eating episodes but without compensatory behaviours. Sub-threshold forms of these disorders, manifesting with only some symptoms of the full syndromes, also cause significant suffering [4]. An epidemiological study has shown that, in Switzerland, the lifetime prevalence rates of these disorders, calculated by adding lifetime prevalence estimates for anorexia nervosa, bulimia nervosa, binge eating disorder, any eating disorder, sub-threshold binge eating disorder and any binge eating, were 17.5% for women and 7.8% for men [5]. According to the authors of this study, these prevalences were comparable to those of other westernised countries and even higher for bulimia nervosa, which is a pathology strongly influenced by culture [6].

Mental disorders frequently develop in adolescents or young adults, and interfere with the developmental tasks required during these periods [7]. These disorders are likely to become established in adulthood, which is why public health initiatives aimed at youth can greatly contribute to the health of the community [7]. Regarding the field of eating disorders more specifically, people with eating disorders often feel ashamed of their disorder, which prevents them from seeking help [8]. This delays the possibility of treatment whereas early intervention improves treatment outcomes [9]. The distress and functional impairment caused by eating disorders, together with eating disorders correlation with depression, anxiety, substance use disorders and elevated risks of suicide attempts, reinforce the need of intervening with prevention interventions before eating disorder onset [1,10,11]. Moreover, there is a lack of treatment facilities providing specialised and evidence-based treatments for eating disorders [12], and evidence-based treatments do not suit every patient, with remission rates not superior to 50–60% of the patients with bulimia nervosa or binge eating disorder [13]. Finally, the costs generated by these disorders are underestimated; because they are not systematically declared, it is indeed difficult to have an objective view of the missed working days or of the complications that they cause. The critical period during which eating disorders develop, the difficulty of treating these disorders, the suffering they cause, together with the costs they generate, highlight their public-health significance, and lead researchers to recommand the development and evaluation of prevention interventions for eating disorders targeting young adults [14].

**Fig 1:**
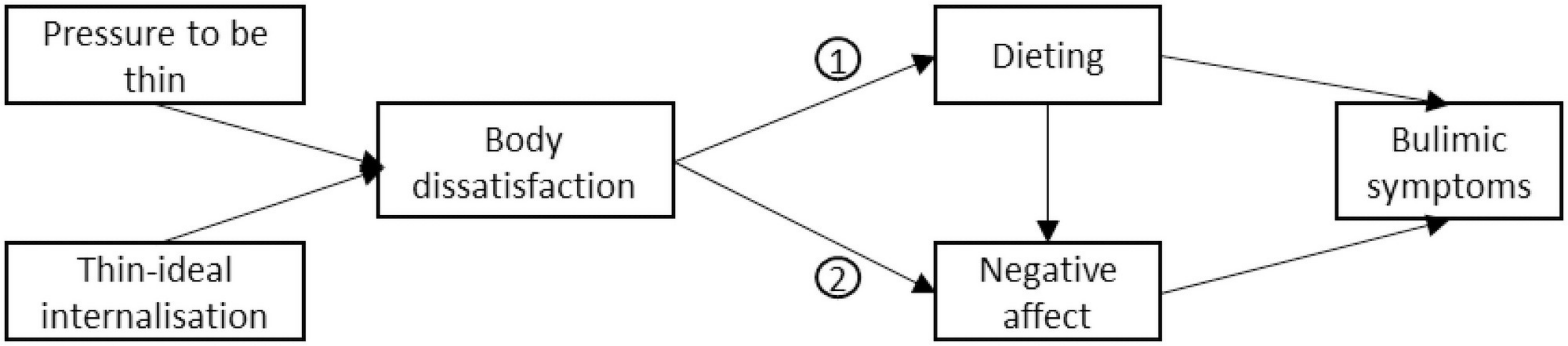
Stice’s Dual Pathway Model.

The prevention interventions built on the Dual Pathway Model [15] as theoretical foundations are among the most effective [16,17]. Stice proposed this model after a meta-analysis of the risk factors leading to compulsive disorder type [18]. It includes two identified pathways leading to the development of compulsive eating disorders: 1) dieting and 2) negative affect (Fig 1), with body dissatisfaction as the necessary and common risk factor.

Based on this model, Stice and colleagues conceptualised a prevention intervention that targets the internalisation of the societal beauty ideal of thinness (thin-ideal internalisation in the model in Fig 1) in order to intervene upstream of body dissatisfaction. This intervention, called “Body Project” (BP), was first evaluated in comparison with a control group without intervention, in female undergraduates aged 18–22, with body dissatisfaction (N = 30), in the United-States (US) [19]. Then, the BP was compared to a placebo intervention specially designed for the study: the “Healthy Weight Program” (HW) in US young women aged 17–29 with body dissatisfaction [20]. When the two interventions were compared, the BP participants showed significant decreases in thin-ideal internalization, body dissatisfaction, dieting, negative affect, and bulimic symptoms. In parallel, decreases in thin-ideal internalization was not observed the HW participants, however this placebo intervention produced significant improvements, albeit smaller, of the other outcomes variables. To clarify these unexpected findings, a four-armed study compared the two intervention programmes with an emotional writing condition and a control group without intervention and followed up for up to three years in 481 US adolescent girls [21,22]. The results were favourable to the BP and the HW compared to the two control conditions, with a reduction in the onset of eating disorders at three years of 60% for the BP and 61% for the HW.

Since then, these two prevention interventions have been evaluated in various contexts. The BP intervention provided greater decreases of eating disorders risk factors than a video-control condition, whether led by a clinician, peers or delivered by an Internet platform [23]. The peer-led version of the BP turned out to produce the greatest reduction of eating disorders onset after four years, compared to the other form of program delivery [24]. The BP intervention was evaluated in other cultural context than US, for example Brazil [25] and Sweden [26], with similar outcomes, and without the need for cultural adaptation [25]. The BP and the HW interventions were also adapted and proposed to women athletes [27]. Both interventions significantly decreased eating disorders risk factors in this population, and the qualitative results suggested the athletes seemed to prefer the HW intervention. The BP is the eating disorder prevention programme which has collected the largest number of multiple independent replications of trials assessing its efficacy and effectiveness in reducing eating disorder risk factors and symptoms [28]. Because of the strong empirical support garnered by the BP in the US, scholars have recommended the examination of its potential to reduce eating disorders onset in Europe as well, which is in need of efficacious eating disorder prevention intervention [29]. Replication trials to assess the effectiveness of the HW, which is the only programme who has successfully addressed the prevention of eating disorders and obesity, are also needed [29].

Most studies evaluated the BP and the HW interventions in a context of selective prevention, intended for people with risk factors. Therefore studies included young women with body dissatisfaction. The BP and the HW interventions are both aimed at older adolescents and young adults, typically students. Excessive concerns with body image and disturbed eating behaviours often appear in adolescence and usually have not been addressed due to many reasons, from a desire to manage the problem by oneself, to a lack of time, or a lack of availability of treatment facilities [30–32]. But when young people arrive at university or enter higher education, the stress due to academic competition, the new environment, and the empowerment that accompanies this new stage of life may increase already existing difficulties [33]. Therefore, young women who are at-risk because of body dissatisfaction could greatly benefit from targeted interventions.

To our knowledge, no study has been carried out in Switzerland to evaluate an intervention for the prevention of eating disorders. Public health in French-speaking Switzerland lacks programs based on evidence in this field. This protocol presents a preliminary study to evaluate the acceptability and the effectiveness of the BP and HW interventions for female students in French-speaking Switzerland. Both of them have produced significant effects. The BP have produced larger effects [21,22], however the HW might be better accepted by certain sub-groups, such as athletes [27]. This is why we are interested in both of them. The study is preliminary in the sense that we want to collect participants’ satisfaction with each of the two interventions in order to assess their acceptability. A French version of the BP already exists, but no studies has yet been published on its effectiveness. The HW program will be translated in French by us. The script will be first translated literally and then adapted according to the Swiss recommendations for a healthy nutrition and physical activity. However, the core of the intervention, which is to set small objectives to modify permanently one’s dietary intakes and physical activity in order to reach energy balance, and discuss their attainment in group, will be kept identical. Besides, the two interventions will be compared to a waiting list to assess the interventions effectiveness, with a power able to detect large effect size.

The goal of this study is to assess the following three hypotheses:

1. The acceptability of the two interventions, assessed by the number of sessions that the participants attend, their satisfaction with the intervention, and qualitative comments provided by the participants, will be good for both interventions;
2. The two interventions BP and HW will improve body dissatisfaction (primary outcome) as well as thin-ideal internalisation, dietary restraint, negative affect, and eating disorder psychopathology (secondary outcomes), compared to a waiting list;
3. The improvements observed in the intervention groups (BP and HW) will be maintained after one month of follow-up.

## Materials and methods

### Study design

**Fig 2:**
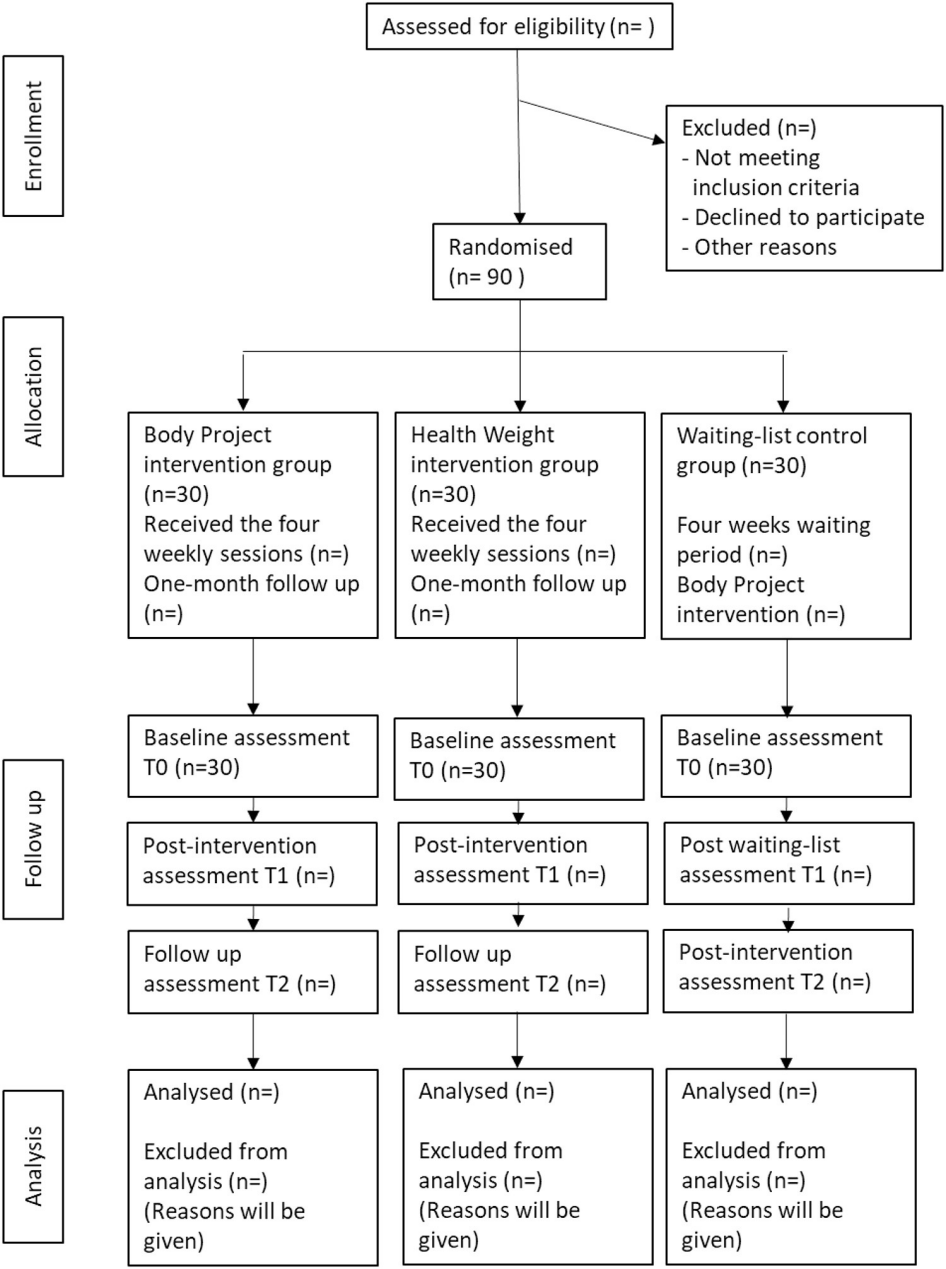
Flowchart illustrating recruitment and assessment of the participants through the study. Adapted from the CONSORT 2010 flow diagram.

To compare the BP and HW interventions to a waiting list, we plan to carry out a three-arm randomised controlled study, including female students from French-speaking Switzerland. We will recruit 90 participants over a 12-month period, starting in January 2021. Participants will be randomly assigned to one of the three arms of the study. Both interventions are composed of four weekly sessions and last one month. The waiting list duration will be set to one month in a parallel way. Participants will be evaluated before (T0) and after (T1) the interventions or the waiting list. The interventions are given to groups of six participants and will start each time a group is formed following randomisation. Following the interventions, the participants will have one month of follow-up without contact with the investigators before a final evaluation (T2). To maximise the feasibility of the study regarding funding and university calendar, participants in the waiting list group will receive the BP intervention following the one-month waiting period instead of waiting until the end of the follow-up assessments. They will be evaluated at the end of the intervention (T2). The procedure is described in Fig 2.

### Participants

The target population of the study are female students from the University of Geneva and other French-speaking cantons, and the University of Applied Sciences and Arts Western Switzerland (HES-SO) aged 18 to 25, with body dissatisfaction and a BMI between 18.5 and 30 kg/m^2^. To recruit the participants, a brief video proposing to test an intervention to improve one’s body dissatisfaction will be posted on the universities’ social networks. Students’ associations will be harnessed to convey information on the study.

The inclusion criteria are as follows:

- Female student;
- Aged between 18 and 25 years old;
- BMI between 18.5 and 30 kg/m^2^;
- French-speaking (or understands French sufficiently to participate in the intervention) and living in Switzerland for at least six months;
- Suffers from body dissatisfaction and obtains a score of at least 26 on the BSQ-8C [34], which represents a moderate concern with body image;
- Accepts the use of a collaborative platform to participate in the intervention group, which implies that her name may be revealed, and accepts that the sessions will be recorded.

The exclusion criteria are:

- Eating disorder diagnosis declared according to the DSM-5 diagnostic criteria (past or current);
- Any diagnosis of mood disorder or anxiety disorder;
- Pregnancy.

These criteria will be assessed during a first meeting via a phone call or videoconference by one of the members of the project team. If the participant meets the inclusion criteria and shows interest, she will receive the information and consent forms by mail, to be signed and returned. The participants will never be met face to face.

### Withdrawals

In the event that a participant develops an eating disorder during the study, she will be withdrawn from the study and referred to a specialised centre. Unhealthy attitudes and behaviours will be carefully monitored during the interventions by the two facilitators who lead the group sessions, who have both a clinical experience in the field of eating disorders and obesity. If the participant becomes pregnant during the study, she will be excluded, since changes in the body as well as hormonal changes, even in early pregnancy, can influence the results of the questionnaires [35].

### Random allocation

After receiving the consent form, the participant will be randomised into one of the three study arms, with a 1: 1: 1 allocation ratio. Randomisation will be blocked to ensure that groups are of equal size and that groups of six participants for each arm are regularly formed. The blocks will be of variable size (3, 6, 9) to protect the concealment. There is no stratification. The randomisation sequence will be implemented in the REDCap software, so none of the team members will know in advance which arm the next participant will be assigned to.

### Sample size

To assess the effectiveness of each intervention, we will recruit a sample of 30 participants per arm, for a total N of 90 participants randomised into three groups. This sample size appears sufficient to detect a large pre-post effect on body dissatisfaction (primary outcome) according to data reported in previous studies [36], and including a hypothetical dropout rate of 10%. The effect sizes (Cohen’s d) reported in the Stice studies are large for the BP or the HW and generally exceed 0.50 (e.g. ref. [36] where the effect sizes were 0.90). To capture a pre-post effect of 0.50 with a power of 0.80 and a probability of 0.05, a sample of 27 people is needed for an intervention (calculated with G * Power 3.1 [37]).

### Interventions

Because of the pandemic situation due to COVID-19, we chose to deliver the interventions virtually via a collaborative platform, in order to keep a group format while preserving a safe distance. To our knowledge, the effectiveness of the BP when delivered virtually has been evaluated by only one Swedish study, the results of which have just been published [26]. The virtually delivered BP intervention preserved its preventive effect in relation to symptoms of eating disorders, restraint, body dissatisfaction, and internalisation of the thin ideal, compared to a placebo condition of expressive writing and a waiting list control condition.

The BP offers discussions and exercises to make participants realise the cost of pursuing social ideals valuing thinness. The exercises proposed in the BP intervention produce cognitive dissonance [38], which is a cognitive discomfort due to a contradiction between speech and beliefs. This discomfort motivates the person to change their beliefs. The curriculum includes discussions on techniques of the advertising industry and the unique ideal of beauty promoted worldwide, as well as on the costs of pursuing the thin ideal. The participants are also challenged to try behaviours they avoid because their appearance bothers them. Societal activism actions are also discussed. The content of the sessions are manualised and can be downloaded from the website www.bodyproejctsupport.org/resources/materials (4-session French Translation).

The HW allows participants to make personalised changes in their diet and physical activity gradually each week to aim for a health ideal rather than a thin ideal. The programme targets small changes, which are based on behavioural change techniques. Advice is given to participants on what types of changes to introduce to promote their health and how to implement changes sustainably. Difficulties are discussed in the group with a problem-solving approach. The last session introduces a long-term view of lifestyle change. The Healthy Weight programme is manualised and the 4-session script can be downloaded from the website: https://healthyweightsupport.weebly.com/healthy-weight.html.

The two interventions include four 90 minutes sessions given over a month by two facilitators, the principal investigator (IC) or the co-investigator (SBDT), and two research assistants. IC and a research assistant will lead all BP sessions and SBDT and the second research assistant will lead all HW sessions. The four facilitators were trained to the BP programme by the person who translated the programme into French. They trained themselves on their own to the use of the HW programme. Before starting the study, a dry run of both programmes will be conducted. The sessions will be held on a collaborative platform in groups of six people and will be recorded. The aim of the recordings is to serve as a means of quality control regarding the way in which the interventions are delivered. The quality control will be carried out internally, on randomly selected portions of sessions. The participants will have signed a consent form for the recording of the sessions. No health or confidential data will be discussed during interventions, which makes it acceptable to record sessions. These records will be destroyed once the quality control has been carried out.

To increase participants’ adherence, reminders will be sent to them via e-mails between sessions.

The waiting list will consist of two assessments, each separated by a one-month interval. Following this waiting time, participants will receive the BP intervention because it is the intervention that has achieved the larger effects [21,22]. A third assessment will take place at the end of the intervention.

### Blinding

The participants or the people performing the interventions will not be blinded to their conditions. In order to minimise bias, the main statistical analyses aimed at evaluating the hypotheses will be performed by a statistician who is blinded to the participants’ allocation.

### Measures

Participants will complete the following questionnaires before the interventions or the waiting list (T0), after the interventions or the waiting list (T1), and after one month of follow-up or intervention (T2).

#### Primary outcome

Body dissatisfaction will be assessed with a short version of the *Body Shape Questionnaire* (BSQ-8C) [34,39], which includes eight items, such as "Have you felt excessively large or rounded?" to be rated on a 6-point Likert scale from 1 *never* to 6 *always*. This instrument has been widely used in research on eating disorders and has been validated in English as well as in French [40].

#### Secondary outcomes

Thin-ideal internalisation will be assessed with the *Socio-Cultural Attitudes Towards Appearance Questionnaire* (SATAQ-4) [41], which includes five items, such as "I want my body to look very thin", to be rated on a 5-point Likert scale from 1 *strongly disagree* to 5 *strongly agree*. This questionnaire has been validated in English and French [42].

Dietary restraint will be assessed with the Restrained Eating subscale of the *Dutch Eating Behaviour Questionnaire* (DEBQ) [43]. This subscale includes 10 questions, such as “Do you intentionally eat foods that are slimming?” to be rated on a scale from 1 *never* to 5 *always*. The DEBQ has been translated and validated in French [44].

Negative affect will be assessed using the *Hospital Anxiety and Depression Scale* (HAD) [45], which is composed of seven items assessing how often the participant experiences anxiety and seven items assessing how often the participant experiences depression on a scale of 0 *never* to 3 *very often*. The HAD has been validated in French [46,47].

Eating disorder psychopathology will be assessed with the *Eating Disorder Examination-Questionnaire* (EDE-Q) [48], which includes 28 items assessing four essential dimensions of eating disorders: dietary restraint, eating concern, shape concern, and weight concern. These four subscales will be used. A total score can also be calculated, which will be used as an indicator of signs of eating disorder psychopathology. Moreover, the monthly frequency of unhealthy behaviours, such as binge eating or inappropriate compensatory behaviours, and height and weight, will be retrieved from this questionnaire as well. Although the participants should not have eating disorders in order to be included in the study, symptoms of eating disorders are present on a continuum, and it is hoped that the interventions lead to improvement. The EDE-Q has undergone validation studies in English and French [49].

Socio-demographic variables will also be included in the questionnaire, in order to collect age, nationality, time spent in Switzerland, university, and faculty.

#### Acceptability

A satisfaction index for the interventions will be added to the post-intervention questionnaires. It includes four questions, “did you find the intervention useful?”, “did the intervention help you?”, “were the key points of the intervention understandable?”, “did you find the exercises useful?” to be rated on a 5-point Likert scale from 1 *not at all* to 5 *very much so*. The satisfaction index will be calculated as a mean of the four subscales. This index and the number of sessions attended by the participants will be used to assess interventions acceptability.

During the last session of the interventions, we will also collect the participants’ comments on the topics that they have appreciated or disliked, and on the topics that might have seemed odd, to allow cultural adaptations if needed, or improvements, to be made to the interventions. We selected the following questions from the focus group discussion guide used by Jarman et al. [50] to prompt the discussion:

1. Thinking about the programme sessions, what did you like about them? Did any particular activities or moments stood out for you?
2. What did you dislike about the programme sessions? Did any particular activities or moments stood out for you?
3. Did you feel comfortable talking during the sessions? If not, why and how can we overcome this or make you feel more comfortable?
4. What do you think we could do to improve the sessions? Was anything missing?
5. How do you think we should advertise it to other young women?
6. Do you have anything else you would like to say?

The participants’ answers will be transcribed verbatim and analysed with thematic analyses [51].

### Data management and statistical analysis

Participants will receive a code upon entering the study. Coding will start at 001 and continue with +1. The signed consent form will contain the equivalence between the participant’s contact details and their code. All forms will be printed and stored in a locked drawer. The electronic forms will be stored in a protected space, which will be backed up regularly during the study. At the end of the study, the data will be stored in a repository, which guarantees that archiving and data sharing will be performed in accordance with FAIR principles. The study will be internally monitored.

The questionnaires (validated questionnaires, socio-demographic variables, satisfaction indices) will be implemented in the REDCap software [52], hosted at the HES-SO (University of Applied Sciences and Arts, Western Switzerland), which will be used to collect the data. The participant’s e-mail address will need to be entered in the platform to allow the surveys to be sent, but it will be coded as an identifier and not exported to the database. Only the participant’s code will represent the participant in the final database.

Missing data will be handled with a single imputation method. To test the hypothesis stipulating a pre-post difference between the participants of the two intervention groups and the participants of the control list, analyses of variance (ANCOVA analyses of covariance with time pre in covariate) will be conducted in order to compare the three groups BP, HW, and waiting list on the different dependent variables, with post-hoc analyses to compare the groups two by two. The primary outcome is body dissatisfaction assessed with the BSQ-8C. Similar analyses will be carried out for the secondary outcomes. Regarding the stability of the intervention and the hypothesis predicting that the observed effects will be maintained after one month of follow-up, repeated measures analyses of variance will be performed. The statistical analyses will be carried out with completers and intent-to-treat.

### Ethics

The study was approved by the Ethics Committee of Geneva, Switzerland (project ID 2020–01010, version 3.0 approved on July 14^th^, 2020) and is registered in the Swiss National Clinical Trial Portal (SNCTP000003978), as well as in ClinicalTrials.gov (NCT04558073). Participants will receive a detailed information sheet by mail explaining the study goals, procedures, and potential risks and benefits. They will be informed that participation in the study is voluntary and that they can withdraw at any time without impacting the course of their university studies. After they have had sufficient time to review this information, they will sign a written consent form, which they will mail back to the study investigators. This consent will be obtained before any procedure is initiated. There is no financial compensation for participating in this study. One month after the last assessment, all participants will be proposed an individual debriefing session via videoconference, in order to close their participation and ensure they do not need any referral to a consultation centre.

## Discussion

This study will provide initial results on the acceptability and effectiveness of the BP and HW interventions in French-speaking Switzerland. These results will be disseminated through scientific articles and at conferences on eating disorders and body image. In order to disseminate the results in the community, we will communicate them to the two main French-speaking websites dedicated to adolescents and young adults health promotion. Potential cultural adaptations that might emerge from the participants’ comments will be considered and discussed with the designers of the interventions, before being adopted. We do not expect major adaptations, since at least the BP programme was used successfully in other cultures than the US without fundamental change [25,26]. In addition, these preliminary data will be used as a basis to write a grant proposal for national funding agencies for investigating the mediating or moderating factors involved in the prevention of eating disorders. In particular, the mechanisms at play in the HW are not yet well understood [53]. An analysis of the stability of the effects of interventions, as well as their effect on the development of eating disorders, requires a larger sample and a longer follow-up period.

This preliminary study will include limitations. A truly "preventive" effect in relation to the onset of an eating disorder will not be appreciable, as longer-term measures are needed. Therefore, we use a "proxy", that is, the measure of a risk factor, which is body dissatisfaction in this case. The disclosure that both programmes should aim at reducing body dissatisfaction could impact the outcomes, however this disclosure is deemed necessary to recruit participants with a sufficient level of body dissatisfaction. Then, this preliminary study will include a sample size that allows the detection of only large effects. We will not assess the stability of the interventions after one month. This study will include only female participants, as the majority of the studies on the BP and HW interventions [17]. Few studies have been conducted on mixed groups, and the results obtained have been less convincing, with young women benefiting less from the effects of interventions when young men were included (e.g. [54]). However, this is as concern because young men also experience body dissatisfaction with impact on their health [55]. Among the strengths of this study, the sample size needed to show a pre-post effect was calculated, which should ensure that we are able to detect a large effect size. Both interventions have shown positive effects in other countries (e.g. [25,26]). Both interventions have specific features, which is why we are evaluating them both. The study design makes it possible to assess their effect in comparison to no intervention in a community setting.

## Conclusion

This study is a first step into the field of selected prevention of eating disorders in Switzerland, where eating disorders are highly present and evidence-based prevention interventions are still lacking.
